# Meta-analysis of the efficacy of proton pump inhibitors for the symptoms of laryngopharyngeal reflux

**DOI:** 10.1590/1414-431X20165149

**Published:** 2016-07-04

**Authors:** C. Liu, H. Wang, K. Liu

**Affiliations:** 1 Department of Otolaryngology, Ji'ning First People's Hospital of Shandong Province, Ji'ning, Shandong, China; 2 Department of Pediatric Dentistry, Ji'ning Stomatological Hospital of Shandong Province, Ji’ning, Shandong, China; 3 Department of Otolaryngology, Ping'du People's Hospital of Shandong Province, Ping'du, Shandong, China

**Keywords:** Gastroesophageal reflux disease, Adult, Proton pump inhibitors, Meta-analysis

## Abstract

The objective of this study was to perform a systematic review and meta-analysis to assess the effectiveness of proton pump inhibitors (PPI) for reflux disease in adult patients with laryngopharyngeal symptoms. A comprehensive search of Cochrane Library, EMBASE, Ovid EBM Reviews, and PubMed was performed for English-language literature about laryngopharyngeal reflux (LPR), in September 2014. The papers were filtered using pre-defined inclusion and exclusion criteria. Eight papers were identified and included in this meta-analysis. The sample comprised a pooled total of 370 patients, of which 210 and 160 patients took PPIs and placebo, respectively. The difference between PPIs and placebo groups in overall improvement of symptoms in adult patients with LPR was not statistically significant (RR=1.22; 95%CI=0.93-1.58; P=0.149). The difference in cough improvement was also not significant between PPIs and placebo groups (RR=0.65; 95%CI=0.30-1.41; P=0.279).

## Introduction

Laryngopharyngeal reflux (LPR) is the retrograde flow of gastric contents into the pharynx and larynx, causing a variety of symptoms. LPR has become a significant and increasingly prevalent disease seen in the otolaryngologist's office. The prevalence of LPR in outpatients is about 10% in the USA (1).

Most of the patients with LPR do not complain of heartburn and regurgitation, which are the classic gastroesophageal reflux disease (GERD) symptoms, and many studies have shown an association between GERD and LPR symptoms. The mechanisms for GERD-associated LPR are considered to be the acid stimulation of vagal afferent nerves and the direct laryngeal contact with gastroesophageal reflux (2). Compared with the esophageal mucosa, the mucosa of the pharynx and larynx are less resistant to the gastric acid effects (3). Small amounts of acid substance is possibly insufficient to cause esophageal symptoms, but may be sufficient to cause laryngeal symptoms.

As LPR is one of many extra-esophageal manifestations of GERD, medical treatment for reflux disease is recommended for LPR. The most common class of drugs prescribed for LPR is the proton-pump inhibitor (PPI), which has shown to benefit patients with LPR in some studies (4). However, most of the studies address empiric therapy, with few randomized, placebo-controlled trials (RCTs) addressing LPR therapy. The aim of the study was to conduct a meta-analysis in order to evaluate the effectiveness of PPI therapy in adult patients with LPR.

## Material and Methods

### Search strategy

A comprehensive search was done using Cochrane Library, EMBASE, Ovid EBM Reviews, and PubMed for English-language literature in September 2014. The following key words were used as search items: laryngeal reflux, pharyngeal reflux, laryngopharyngeal reflux, laryngopharyngeal reflux disease, laryngopharyngeal reflux diseases, LPR, LPRD, reflux laryngitis, reflux pharyngitis, hoarseness, throat clearing, throat mucus, postnasal drip, dysphagia, cough, dyspnea, dyspnea, globus, throat lump, rumination, vocal cord/fold edema, posterior laryngitis, vocal cord/fold granuloma, gastric aspiration(s), gastric regurgitation(s), extraesophageal reflux, extraesophageal reflux disease, gastropharyngeal reflux, GPR, proton pump inhibitor(s), PPI, proton pump antagonist, proton pump blocker, omeprazole, lansoprazole, pantoprazole, rabeprazole, esomeprazole, acid suppressive therapy, and anti-reflux therapy.

Inclusion criteria: 1) patients with laryngeal or pharyngeal reflux lasting ≥2 weeks; 2) adult patients aged ≥18 years; 3) studies comparing PPIs and placebo interventions; 4) study personnel, clinicians and patients were blind to the treatment; 5) curative effect criterion; 6) randomized controlled trials (RCTs) or controlled clinical trials.

Exclusion criteria: 1) patients with laryngeal or pharyngeal reflux lasting <2 weeks; 2) children; 3) study without curative effect criterion; 4) single-/multi- intervention; 5) presence of several diseases; 6) duplicate publications; 7) reviews, case reports, single clinical trials, and expert opinions.

All titles and abstracts of the studies were reviewed, and the full text of the eligible studies was obtained for further review. The bibliography of the selected literature was reviewed to determine whether any relevant study had been missed.

### Quality assessment

The level of evidence of the included literature was graded according to Oxford Centre for Evidence-Based Medicine 2011, as follows: level 1: systematic review of randomized trials or n-of-1 trials; level 2: randomized trial or observational study with dramatic effect; level 3: non-randomized controlled cohort/follow-up study; level 4: case-series, case-control studies, or historically controlled studies; level 5: mechanism-based reasoning.

## Results

**Figure 1:**
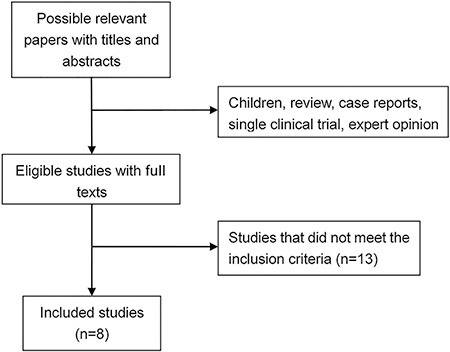
Study selection process.

The systematic search strategy produced 2420 possibly relevant English-language papers. Only 21 studies meeting the inclusion criteria were selected and their full texts obtained for further review. After reviewing the full texts, 8 papers (5-12) were identified and included in our analysis (Figure 1). Of the 8 studies, 7 were placebo-controlled, and 1 was a placebo-controlled, cross-over trial. Table 1 shows the characteristics of the studies included in the meta-analysis.

**Table:**
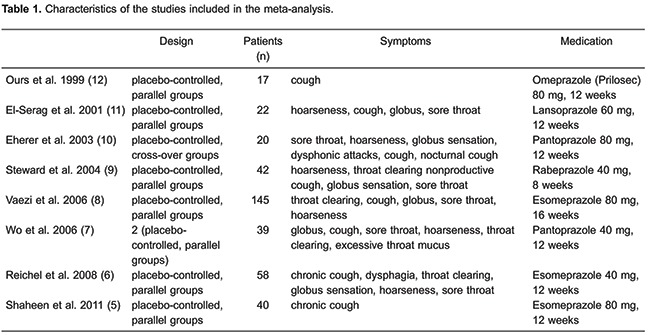

**Figure 2:**
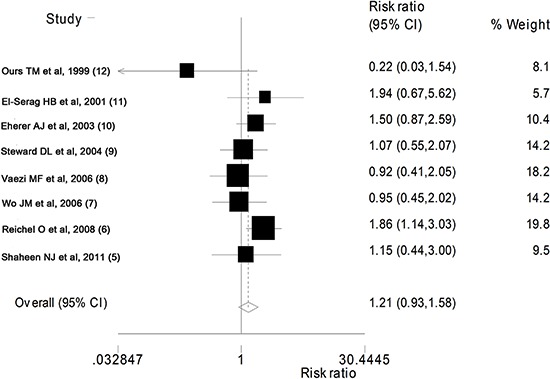
Forest plot showing the effect of PPIs on improvement in LPR symptoms. See Table 1 for reference details.

The total number of patients included in our analysis was 370, of which 210 and 160 patients took PPIs and placebo, respectively. As shown in Figure 2, the pooled effect of the difference between the effect of PPIs and placebo treatment in overall improvement of symptoms in adult patients with LPR was not statistically significant (RR=1.22; 95%CI=0.93-1.58; P=0.149).

**Figure 3:**
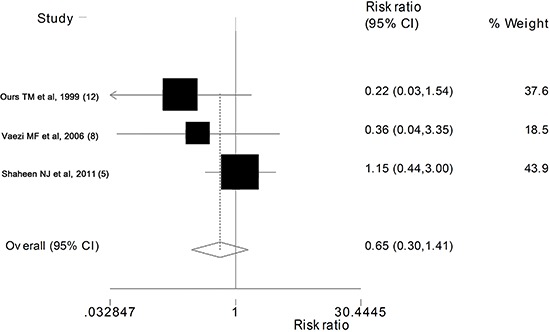
Forest plot showing the effect of PPIs on cough improvement. See Table 1 for reference details.

Of the 8 studies, 3 contained data on cough improvement. The pooled effect analysis shows that the difference between PPIs and placebo groups in cough improvement was not statistically significant (RR=0.65; 95%CI=0.30-1.41; P=0.279) (Figure 3).

## Discussion

The results of the meta-analysis showed that PPIs therapy had no significant advantage in improving or resolving LPR symptoms over placebo. Similarly, no significant difference between the PPIs and placebo was found towards cough improvement.

One of the 8 studies was a randomized controlled crossover trial (10), all other studies were randomized controlled trials. All patients were randomized into PPIs or placebo treatment groups. This is a critical issue regarding the quality of the present meta-analysis. The limitation is that different types of PPIs were used in the studies. The PPIs included omeprazole (12), lansoprazole (11), pantoprazole (7,10), rabeprazole (9), and esomeprazole (5,6,8). The daily dose of PPIs varied from 40 to 80 mg lasting at least 8 weeks, which was higher than that routinely used in GERD patients (13).

Most clinicians recommend PPIs for the treatment of LPR, and are convinced of their benefits. However, our study shows that PPIs have a similar effect as placebo in the improvement of LPR symptoms. The various symptoms of LPR include hoarseness, cough, globus, sore throat and dysphagia, which have been used as labels for the reflux symptom index (RSI) (14). The RSI and reflux finding score (RFS) (15), used in one study in our meta-analysis (6), have been widely used in evaluating LPR. The diagnosis of LPR is mainly dependent on symptom evaluation. In this study, we analyzed the improvement of symptoms in adult patients with LPR taking PPIs or placebo. Our results might have been different if symptoms were evaluated using the RSI and/or RFS scores.

Two previous meta-analysis evaluated the efficacy of PPIs for GERD-related laryngeal symptoms (16,17 [Bibr B18] [Bibr B19]). Gatta et al. (16) reviewed 4 RCTs (8,9,11,12) and 1 randomized clinical control trial (10) in 2007, and found no difference between the effects of PPI therapy and placebo in laryngopharyngeal symptoms improvement or resolution. Qadeer et al. (17) reviewed 8 studies (7,8,) in 2006 and reported that PPI therapy may offer a modest, but non-significant clinical benefit over placebo in GERD-related chronic laryngitis. Our study also found no difference in the effect of PPI therapy and placebo in the improvement of symptoms in adult patients with LPR.

PPI therapy was found effective for treating LPR in one study (20), and BID (twice daily) PPI appeared to be more effective than QD (once daily) PPI (21). In our study, BID PPI was used in seven studies (5,6,), and QD PPI in one study (7).

We found a strong placebo effect, which may have been a result of the scoring performed by the patients. The symptoms of LPR are not correlated with its signs (22,23). Another explanation is a possible over-diagnosis of LPR, which is based on nonspecific symptoms found also in other diseases. Laryngopharyngeal pH testing can be used to define laryngopharyngeal reflux, but it is not used universally and lacks a gold standard criteria. A gold standard for LPR diagnosis should be developed in the future.

Most of the studies included into our meta-analysis had small sample sizes, ranging from 17 to 145 study participants. The small sample sizes could have resulted in Type II statistical errors. Large sample RCTs are needed to estimate the true effect of PPIs.

Our study suggests that PPIs and placebo therapy are similarly effective in improving LPR symptoms in adult patients. A better understanding and further study of the effect of PPIs on LPR is necessary.
